# Integration of C_1_ and C_2_ Metabolism in Trees

**DOI:** 10.3390/ijms18102045

**Published:** 2017-09-23

**Authors:** Kolby J. Jardine, Vinicius Fernandes de Souza, Patty Oikawa, Niro Higuchi, Markus Bill, Rachel Porras, Ülo Niinemets, Jeffrey Q. Chambers

**Affiliations:** 1 Climate Science Department, Earth Science Division, Lawrence Berkeley National Laboratory, One Cyclotron Rd, building 64-241, Berkeley, CA 94720, USA; mbill@lbl.gov (M.B.); rcporras@lbl.gov (R.P.); jchambers@lbl.gov (J.Q.C.); 2 National Institute for Amazon Research, Ave. Andre Araujo 2936, Manaus, AM 69060-001, Brazil; viniciusfernandes11@yahoo.com.br (V.F.d.S.); higuchi.niro@gmail.com (N.H.); 3 Department of Earth and Environmental Sciences, California State University, East Bay, North Science 329, 25800 Carlos Bee Boulevard, Hayward, CA 94542, USA; patty.oikawa@csueastbay.edu; 4 Department of Plant Physiology, Estonian University of Life Sciences, Kreutzwaldi 1, 51014 Tartu, Estonia; ylo@emu.ee; 5 Estonian Academy of Sciences, Kohtu 6, 10130 Tallinn, Estonia; 6 Department of Geography, University of California Berkeley, 507 McCone Hall #4740, Berkeley, CA 94720, USA

**Keywords:** internal recycling of carbon, volatile emissions, central metabolism, plant growth and senescence, methanol, acetic acid

## Abstract

C_1_ metabolism in plants is known to be involved in photorespiration, nitrogen and amino acid metabolism, as well as methylation and biosynthesis of metabolites and biopolymers. Although the flux of carbon through the C_1_ pathway is thought to be large, its intermediates are difficult to measure and relatively little is known about this potentially ubiquitous pathway. In this study, we evaluated the C_1_ pathway and its integration with the central metabolism using aqueous solutions of ^13^C-labeled C_1_ and C_2_ intermediates delivered to branches of the tropical species *Inga edulis* via the transpiration stream. Delivery of [^13^C]methanol and [^13^C]formaldehyde rapidly stimulated leaf emissions of [^13^C]methanol, [^13^C]formaldehyde, [^13^C]formic acid, and ^13^CO_2_, confirming the existence of the C1 pathway and rapid interconversion between methanol and formaldehyde. However, while [^13^C]formate solutions stimulated emissions of ^13^CO_2_, emissions of [^13^C]methanol or [^13^C]formaldehyde were not detected, suggesting that once oxidation to formate occurs it is rapidly oxidized to CO_2_ within chloroplasts. ^13^C-labeling of isoprene, a known photosynthetic product, was linearly related to ^13^CO_2_ across C_1_ and C_2_ ([^13^C_2_]acetate and [2-^13^C]glycine) substrates, consistent with reassimilation of C_1_, respiratory, and photorespiratory CO_2_. Moreover, [^13^C]methanol and [^13^C]formaldehyde induced a quantitative labeling of both carbon atoms of acetic acid emissions, possibly through the rapid turnover of the chloroplastic acetyl-CoA pool via glycolate oxidation. The results support a role of the C_1_ pathway to provide an alternative carbon source for glycine methylation in photorespiration, enhance CO_2_ concentrations within chloroplasts, and produce key C_2_ intermediates (e.g., acetyl-CoA) central to anabolic and catabolic metabolism.

## 1. Introduction

Methanol is the most abundant non-methane volatile organic compound (VOC) in the atmosphere with an array of surface sources dominated by emissions from terrestrial ecosystems [1,2]. While many VOCs produced by plants are highly species-specific (e.g., volatile isoprenoids), methanol production and emission appear to be a universal feature of plants from crops to tropical trees. Modeling studies estimate global plant-derived methanol emissions to the atmosphere (100–128 Tg·year^−1^) vastly exceed anthropogenic emissions [1,2]. Consistent with this view, many land-atmosphere flux studies above agricultural fields [3], managed forests [4], and natural forests [5] have identified methanol as a major, sometimes dominant, component of ecosystem volatile emissions. Given its relatively long atmospheric lifetime of ~10 days, methanol emitted from terrestrial vegetation into the atmosphere can be transported over regional and global scales while participating in a number of photochemical reactions that impact air quality and the oxidative capacity of the troposphere [1,6,7].

Methanol production in plants is largely attributed to changes in chemical and physical cell wall properties associated with the hydrolysis of methyl esters of cell wall carbohydrates like pectin [8,9,10]. Foliar methanol emissions have been shown to tightly correlate with leaf expansion rates [11] and numerous studies have consistently shown that young expanding leaves emit greater amounts of methanol than mature leaves [8,11,12]. Owing to its high solubility in water (Henry’s law constant *H* = 0.461 Pa·m^3^·mol^−1^ at 25 °C), previous experimental and physicochemical emission modeling studies have suggested that changes in stomatal conductance can uncouple instantaneous methanol production rates from emission rates due to an increase in aqueous phase concentrations [13,14]. Nonetheless, these physicochemical mechanisms do not include active metabolism of methanol [13,15]. However, substantial evidence exists that plants are able to metabolize methanol [16,17], giving rise to the possibility that production rates are higher than emission rates. For example, a natural abundance carbon isotope analysis of plant derived methanol suggested the existence of an active methanol consumption pathway leading to an altered carbon isotopic signature of the methanol emitted to the atmosphere [14]. This is consistent with isotopic labeling experiments which revealed that [^13^C]methanol can be incorporated into amino acids such as serine and methionine as well as phospholipids like phosphatidylcholine [17,18]. In addition, feeding leaves with 10% methanol solutions strongly altered the expression of hundreds of genes involved in energy metabolism, cell communication and transduction processes, and cell division and growth [19], further confirming that methanol can be taken up by leaves and profoundly alter metabolism.

Over 50 years ago, gas-phase leaf uptake of ^14^C-labeled methanol, formaldehyde, formic acid, and CO_2_ (up to 1% by volume) was used to demonstrate the existence of an oxidative C_1_ pathway in plants [20]. This pathway was postulated to contribute CO_2_ to photosynthesis as well as to contribute to the production of photorespiratory intermediates, like serine. The mechanism of C_1_ integration into the photorespiratory C_2_ cycle has been confirmed by exposing ^13^C-formaldehyde to leaf discs followed by ^13^C-NMR analysis of photorespiratory intermediates [21]. These results confirmed the postulated hypothesis [22] that formaldehyde can be activated to 5,10-methylene tetrahydrofolate (5,10-CH_2_-THF), a universal donor of methyl groups in biology. Song et al. (2013) suggested that this “alternate” entry point for carbon into the photorespiratory pathway occurs independently of the Benson-Calvin cycle [21]. C_1_ metabolism is thought to be essential for plants not only through its support to photorespiration and photosynthesis, but also through its supply of C_1_ units needed to synthesize proteins, nucleic acids, pantothenate and a large array of methylated metabolites [23]. Although methoxy-groups (functional group or organic compounds constituted of methyl group) of compounds like pectin, lignin, alkaloids, and betaines can make up several percent of plant dry weight [24], little is known about the enzymes, integrated pathways and regulatory mechanisms associated with fluxes of the C_1_ units in plant cells [25]. Although emissions of individual volatile components of the C_1_ pathway (methanol, formaldehyde, formic acid, carbon dioxide) from plants have been observed, emission dynamics of the complete pathway have not been reported. This is in part due to the analytical difficulties in measuring the intermediates of the C_1_ pathway, which are often present in low abundances in the troposphere ranging from several parts per trillion to several parts per billion, and are highly volatile, water soluble and, in the case of formaldehyde, highly reactive. Moreover, C_1_ volatiles in the lower troposphere can arise from a complex mixture of biological and anthropogenic surface emissions and can be produced directly in the atmosphere as intermediates of photochemical oxidation reactions. Therefore, owing in part to the technical difficulties in measuring volatile C_1_ intermediates, the occurrence of the C_1_ pathway among plants and their quantitative importance for central carbon metabolism in natural and managed ecosystems remains unclear.

In this study, we applied highly sensitive and fast instrumentation, developed to investigate the chemistry of volatiles in the atmosphere, to the study of C_1_ metabolism in tropical trees in the central Amazon as part of the Next Generation Ecosystem Experiments Tropics (NGEE Tropics). We applied high sensitivity proton transfer reaction–mass spectrometry (PTR-MS) which uses soft chemical ionization of volatiles through the transfer of protons from hydronium ions (H_3_O^+^). PTR-MS is well suited for the real-time analysis of volatile and reactive intermediates of the C_1_ pathway due to the lack of sample pretreatment and low detection limits in the parts per trillion range [26]. Using dynamic ^13^C-pulse chase experiments, we evaluated the potential existence of the complete C_1_ pathway and its integration with C_2_ metabolism in individual branches of a tropical pioneer species using aqueous solutions of ^13^C-labeled C_1_ (methanol, formaldehyde, formic acid) and C_2_ (acetic acid, glycine) intermediates delivered via the transpiration stream. We hypothesized that CO_2_ produced within chloroplasts through formate oxidation may lead to the incorporation of C_1_ carbon into primary photosynthetic products (e.g., isoprenoids, sugars, fatty acids, etc.) via reassimilation of CO_2_. Given the demonstrated role of the C_1_ pathway in methylation and biosynthesis of metabolites in plants, we hypothesized that the central C_2_ metabolites (e.g., acetic acid and acetyl-CoA) may directly derive carbon from C_1_ intermediates, as has been well demonstrated in methylotrophic bacteria which incorporate C_1_ carbon into serine via the “serine cycle”, resulting in the production of acetyl-CoA/acetate [27]. We tested these hypotheses in *Inga edulis*, an isoprene-emitting legume tree native throughout South and Central America [28], through dynamic pulse-chase experiments with aqueous solutions of ^13^C-labeled C_1_ (methanol, formaldehyde, formate) and C_2_ (acetate, glycine) metabolites fed through the transpiration stream of detached branches together with real-time stable carbon isotope analysis of C_1_ (methanol, formaldehyde, formic acid, CO_2_), C_2_ (acetic acid, methyl acetate), and C_5_ (isoprene) leaf volatile emissions using proton transfer reaction-mass spectrometry (PTR-MS), gas chromatography-mass spectrometry (GC-MS), isotope ratio mass spectrometry (IRMS), and cavity ring down spectrometry (CRDS).

## 2. Results

### 2.1. [^13^C]Methanol Pulse Chase Experiments

**Figure 1:**
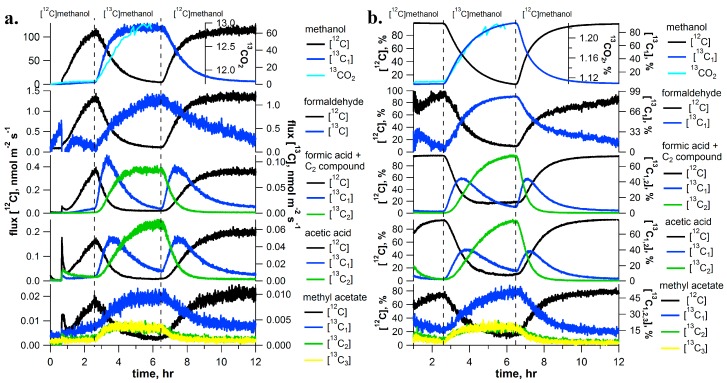
Example [^13^C]methanol pulse-chase experiment on a detached branch of *I. edulis* showing real-time [^13^C]labeling and unlabeling dynamics of C_1_ and C_2_ volatile metabolites using proton transfer reaction–mass spectrometry (PTR-MS) (absolute emissions in (**a**) and relative emissions in (**b**)). Emissions of [^13^C]formaldehyde was estimated by subtraction of the interference by the O_2_^+^ ion at *m*/*z* 32. In addition, ^13^CO_2_ isotope flux through the dynamic headspace atmosphere are also shown up to the end of the [^13^C]methanol labeling period and are based on air sample collections in vials and offline isotope ratio analysis by isotope ratio mass spectrometry (IRMS). Vertical dashed lines indicate a rapid change of solutions delivered to the transpiration stream (i.e., [^12^C]methanol to [^13^C]methanol and vice versa).

To trace the metabolism of methanol in *I. edulis* trees, detached branches were placed in solutions containing either methanol or [^13^C]methanol while real-time ^13^C-labeling analysis of CO_2_ and volatile emissions from the leaves were studied using PTR-MS and GC-MS, together with IRMS or CRDS. Immediately upon placing the detached branch in the methanol solution, a strong increase in emissions of the C_1_ volatiles methanol, formaldehyde, and formic acid was detected (Figure 1). Simultaneously, a strong stimulation of the C_2_ volatiles acetic acid and methyl acetate emissions were observed (Figure 1).

Upon transfer of the branch into the [^13^C]methanol solution, [^13^C]methanol emissions were observed within minutes and this was associated with a near quantitative replacement of [^12^C]methanol emissions (Figure 1). After several hours in the [^13^C]methanol solution, [^13^C]methanol emissions represented 94% of total methanol emissions. Together with [^13^C]methanol emissions, there was a near complete replacement of [^12^C]formaldehyde emissions with [^13^C]formaldehyde emissions (95% of total).

Although emission of [^13^C]formic acid was expected, strong emissions of an interfering C_2_ compound, tentatively identified as ethanol, was found to dominate the PTR-MS signals for [^12,13^C]formic acid (*m*/*z* 47, 48) (Figure 1). Confirmation of the presence of the C_2_ compound comes from the fact that both carbon atoms ([^13^C_2_]ethanol) become strongly ^13^C-labeled. With an initial rise then fall in the single labeled [^13^C_1_] emissions followed by a replacement with double labeled [^13^C_2_] emissions. Upon placement of the branch in the [^13^C]methanol solution, [^12^C]ethanol declined and it was partially replaced by [^13^C_1_]ethanol which reached up to 45% of total emissions. However [^13^C_1_]ethanol was subsequently replaced by [^13^C_2_]ethanol emissions which reached 75% of total ethanol emissions after several hours in the [^13^C]methanol solution. Although emissions of [^12,13^C]formic acid could not be confirmed due to the strong emissions of the interfering C_2_ compound (presumably ethanol), significant emissions of ^13^CO_2_ were determined by both IRMS and CRDS, suggesting the production of formate from labelled methanol. For example, a time series of air samples collected in glass vials followed by CO_2_ isotopic analysis by IRMS confirmed branch emissions of ^13^CO_2_ which also increased and then stabilized together with [^13^C]methanol emissions (Figure 1).

Similar to the pattern observed for ethanol emissions during [^13^C]methanol labeling, both carbon atoms of the acetic acid emissions were quantitatively labeled. Initially, [^12^C]acetic acid emissions were replaced by [^13^C_1_]acetic acid emissions which reached 40% of total emissions after one hour. However, [^13^C_1_]acetic acid emissions were subsequently replaced by [^13^C_2_]acetic acid emissions, which reached 78% of total emissions after several hours in the [^13^C]methanol solution. Likewise, [^13^C_1_]methyl acetate emissions increased to 53% of total emissions and significant labeling of [^13^C_2,3_]methyl acetate was observed. Thermal desorption GC-MS was used to determine the distribution of the ^13^C-label in the methoxy versus the acetate group of methyl acetate emissions (Supplementary Figure S1a). This analysis revealed that the methoxy group of methyl acetate was most strongly ^13^C-labeled, with [^13^C_1_/^12^C] ratio of the methoxy group up to 76%. Moreover, the acetate group also showed significant labeling with [^13^C_1_/^12^C] and [^13^C_2_/^12^C] ratios reaching values up to 4–5% with a stronger ^13^C-labeling of ^13^C_2_ acetate group relative to [^13^C_1_]acetate. Thus, [^13^C_1_]methyl acetate was the most abundant isotopologue emitted and [^13^C_3_]methyl acetate was more abundant than [^13^C_2_]methyl acetate. While the methoxy group was more strongly labeled than the acetate group under the [^13^C]methanol, the [^13^C_3_/^12^C] ratio of methyl acetate reached values up to 15%. Thus, a substantial fraction of methyl acetate was emitted as fully ^13^C-labeled ([^13^C_3_]methyl acetate). The results show that under the [^13^C]methanol labeling conditions used, near complete carbon replacement was observed in emissions of C_1_ (methanol and formaldehyde), C_2_ (ethanol, acetic acid, and the methoxy group of methyl acetate). A similar percentage of carbon replacement for formic acid emissions was expected, but could not be determined due to the PTR-MS interference of a C_2_ compound (ethanol). In contrast, CO_2_ (Figure 1), the acetate group of methyl acetate (Figure 1 and Supplementary Figure S1a), and isoprene (see Section 2.6), showed only partial carbon replacement, suggesting multiple carbon sources.

Upon re-inserting the branch back into the [^12^C]methanol solution, a rapid unlabeling of the C_1_ and C_2_ volatiles occurred within minutes. During this process, [^13^C]methanol, [^13^C]formaldehyde, and ^13^CO_2_ emissions were quantitatively replaced by [^12^C]methanol, [^12^C]formaldehyde, and ^12^CO_2_ emissions. Moreover, [^13^C_2_]ethanol, [^13^C_2_]acetic acid, and [^13^C_1–3_]methyl acetate were replaced by [^12^C]ethanol, [^12^C]acetic acid, and [^12^C]methyl acetate. As a reversal of the labeling process, the [^13^C_2_] volatile was first replaced by [^13^C_1_] emissions which were subsequently replaced by [^12^C] emissions.

### 2.2. [^13^C]Formaldehyde Pulse-Chase Experiments

**Figure 2:**
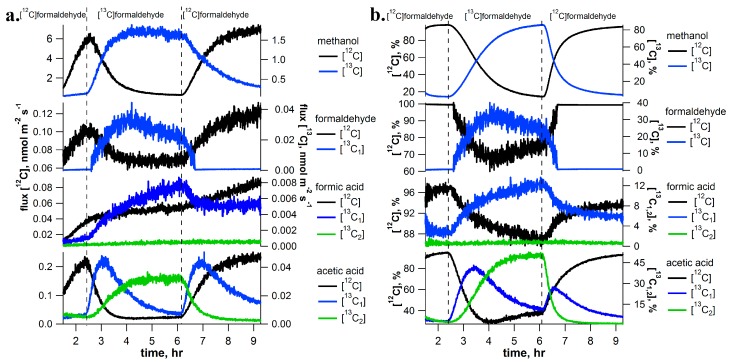
Example [^13^C]formaldehyde pulse-chase experiment on a detached branch of *Inga edulis* showing real-time [^13^C]labeling and unlabeling dynamics of C_1_ and C_2_ volatile metabolites using PTR-MS (absolute emissions in (**a**) and relative emissions in (**b**)). Emissions of [^13^C]formaldehyde was estimated by subtraction of the interference by the O_2_^+^ ion at *m*/*z* 32. Vertical dashed lines indicate a rapid change of solutions delivered to the transpiration stream (i.e., formaldehyde to [^13^C]formaldehyde and vice versa). Note that although isotopic analysis of CO_2_ emissions were not available during [^13^C]formaldehyde labeling experiments, the appearance of ^13^C-isoprene emissions provides indirect evidence for the production (and reassimilation) of ^13^CO_2_ (see Figure 6).

[^13^C]formaldehyde pulse-chase experiments with detached *I. edulis* branches had similar ^13^C-labeling patterns as those with [^13^C]methanol, but showed several important differences (Figure 2 for a representative example). As observed with methanol feeding, formaldehyde solutions resulted in a strong stimulation of both methanol and formaldehyde emissions. In contrast to methanol solutions, formic acid emissions were detectable without the apparent interference of the C_2_ compound (ethanol) emissions. However, as under methanol solutions, formaldehyde solutions resulted in a clear stimulation of the emissions of the C_2_ compound acetic acid. Upon transition into the [^13^C]formaldehyde solution, a stimulation of [^13^C]methanol (85% total), [^13^C]formaldehyde (70% total), and [^13^C]formic acid (20% total) emissions were observed.

Similarly, 90% replacement of both carbon atoms of acetic acid occurred. First an increase in [^13^C_1_]acetic acid (75% of total emissions) occurred and it was subsequently replaced by [^13^C_2_]acetic acid (90% of total emissions). Upon re-inserting the branch in the [^12^C]formaldehyde solution, unlabeling of the C_1_ and C_2_ metabolites occurred within minutes. During this process, [^13^C]methanol, [^13^C]formaldehyde, and [^13^C]formic acid emissions were quantitatively replaced by [^12^C]methanol, [^12^C]formaldehyde, and [^12^C]formic acid emissions, respectively. In addition, [^13^C_2_]acetic acid was replaced by [^13^C_1_]acetic acid which was subsequently replaced by [^12^C]acetic acid. Although IRMS and CRDS instrumentation was not available during formaldehyde labeling experiments, partial labeling of CO_2_ emissions was anticipated. The production of ^13^CO_2_ under [^13^C]formate solutions is supported by the ^13^C-labeling analysis of isoprene (see and Supplementary Figure S1).

### 2.3. [^13^C]Formate Pulse-Chase Experiments

**Figure 3:**
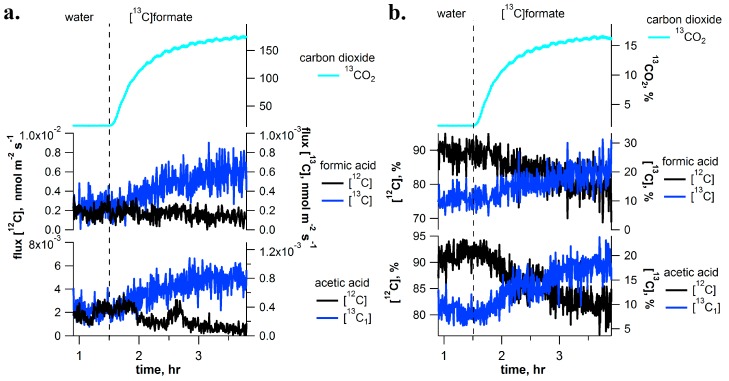
Example [^13^C]formate labeling experiment on a detached branch of *I. edulis* showing real-time [^13^C]labeling and unlabeling dynamics of C_1_ and C_2_ volatile metabolites using PTR-MS (absolute emissions in (**a**) and relative emissions in (**b**)). ^13^CO_2_ branch emission of the dynamic headspace atmosphere are also shown based on cavity ring down spectrometry (CRDS). The vertical dashed line indicates a rapid change of solutions delivered to the transpiration stream (i.e., water to [^13^C]formate). Note, despite a strong labeling of CO_2_ emissions and a detectable labeling of formic and acetic acid emissions, significant labeling of methanol and formaldehyde emissions was not observed (data not shown).

**Figure 6:**
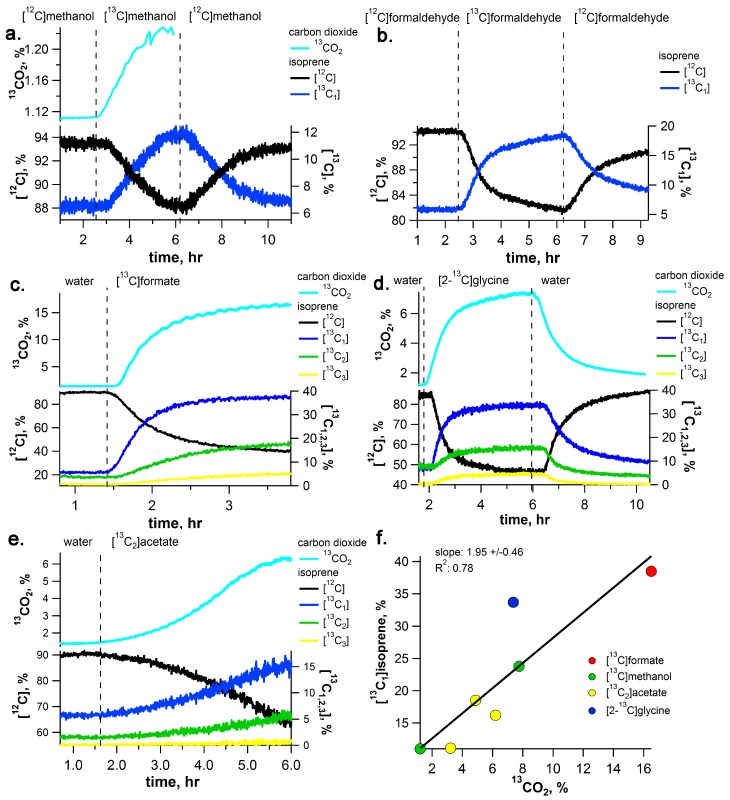
Summary of relationship between the carbon isotopic composition of CO_2_ in the dynamic headspace atmosphere(^13^CO_2_, % total) and the percentage of leaf isoprene emitted as [^12^C]isoprene and [^13^C_1–3_]isoprene during (**a**) [^13^C]methanol; (**b**) [^13^C_2_]formaldehyde; (**c**) [^13^C]formate; (**d**) [2-^13^C]glycine; and (**e**) [^13^C_2_]acetate labeling of detached *I. edulis* branches via the transpiration stream. Also shown in (**f**) is the linear relationship observed between the percentage of ^13^CO_2_ during the labeling experiments and the corresponding percentage of [^13^C_1_]isoprene emissions relative to total isoprene emissions. Note that isotopic analysis of CO_2_ was not available during the [^13^C]formaldehyde labeling experiments.

In the presence of formate dehydrogenase in chloroplasts, we hypothesized that formate may be oxidized to carbon dioxide. Thus, during [^13^C]formate pulse-chase experiments, real-time ^13^C-labeling of CO_2_ emissions were quantified using CRDS together with volatile emissions using PTR-MS (Figure 3). Unlike methanol and formaldehyde solutions, branches fed with [^13^C]formate solutions did not stimulate increased emissions of [^13^C]methanol and [^13^C]formaldehyde (data not shown). However, [^13^C]formate solutions stimulated stronger emissions of ^13^CO_2_ than observed under [^13^C]methanol with ^13^C/^12^C ratios reaching values up to 16.5% compared to 1–7% under [^13^C]methanol (see Figure 3 and Figure 6c, Supplementary Figure S1). In addition, a small but clear ^13^C-labeling of formic acid (8%) and acetic acid (17%) emissions were also observed from the branches in [^13^C]formate solutions. Moreover, isoprene emissions were more strongly labeled under [^13^C]formate than under [^13^C]methanol and [^13^C]formaldehyde solutions. Under [^13^C]formate, [^13^C_1_]isoprene emissions represented 38.5% of total emissions whereas under [^13^C]methanol and [^13^C]formaldehyde, [^13^C_1_]isoprene emissions represented 12–23.8% of total emissions (also see GC-MS labeling analysis of [^13^C_1_]isoprene in Supplementary Figure S1).

### 2.4. [2-^13^C]Glycine Pulse-Chase Experiments

**Figure 4:**
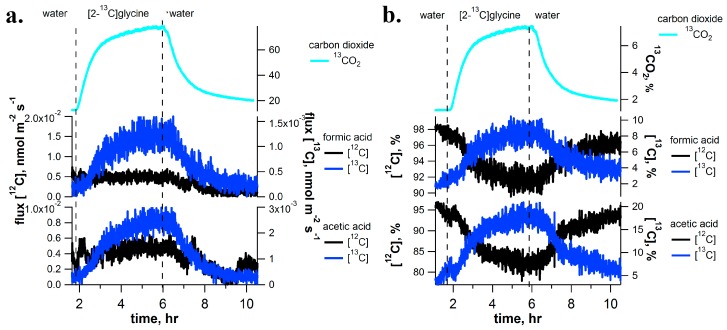
Example [2-^13^C]glycine pulse-chase experiment on a detached branch of *I. edulis* showing real-time [^13^C]labeling and unlabeling dynamics of C_1_ and C_2_ volatile metabolites using PTR-MS (absolute emissions in (**a**) and relative emissions in (**b**)). ^13^CO_2_ branch emissions are also shown based on CRDS. Vertical dashed lines indicate a rapid change of solutions delivered to the transpiration stream (i.e., water to [2-^13^C]glycine and vice verca). Note, despite a strong labeling of CO_2_ emissions, significant labeling of methanol and formaldehyde emissions were not observed (data not shown).

To further explore the connections between the C_1_ pathway and photorespiration, [2-^13^C]glycine (a photorespiratory intermediate) was delivered to *I. edulis* branches while real-time isotope composition of C_1_ and C_2_ volatile emissions was analyzed by PTR-MS and CRDS. Upon transferring the branch from water into a [2-^13^C]glycine solution, volatile ^13^C-labeling patterns (formic acid, acetic acid and isoprene) and CO_2_ (Figure 4 and Figure 6d) were similar to those under [^13^C]formate solutions. Like [^13^C]formate labelling, [2-^13^C]glycine delivery to *I. edulis* branches resulted in small labelling of formic acid and acetic acid emissions with [^13^C]formic acid and [^13^C_1_]acetic acid representing up to 10% and 20% of total emissions, respectively (Figure 4). However, while [^13^C]formate labelling resulted in 16.5% ^13^CO_2_ in the headspace atmosphere, [2-^13^C]glycine labelling resulted in reduced ^13^CO_2_ labelling of 7.4%. As with [^13^C]formate labeling, enhancements in [^13^C]methanol and [^13^C]formaldehyde emissions were not observed (data not shown).

### 2.5. [^13^C_2_]Acetate Pulse-Chase Experiments

**Figure 5:**
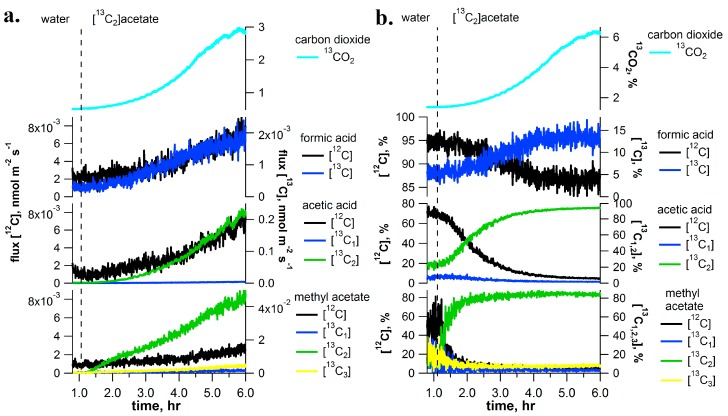
Example of [^13^C_2_]acetate labeling of a detached branch of *I. edulis* showing real-time [^13^C]labeling dynamics of C_1_ and C_2_ volatile metabolites using PTR-MS (absolute emissions in (**a**) and relative emissions in (**b**)). ^13^CO_2_ emission analysis of the dynamic headspace atmosphere are also shown based on CRDS. Vertical dashed line indicates a rapid change of solutions delivered to the transpiration stream (i.e., water to [^13^C_2_]acetate). Note: despite a strong labeling of CO_2_ emissions, significant labeling of methanol and formaldehyde emissions were not observed (data not shown).

To investigate the role of the respiratory substrate acetate in the production of C_1_ and C_2_ metabolites, [^13^C_2_]acetate solutions were delivered through the transpiration stream of detached *I. edulis* branches. Delivery of [^13^C_2_]acetate to leaves resulted in a quantitative replacement of [^12^C]acetic acid emissions with [^13^C_2_]acetic acid emissions (95% of total) (Figure 5). Throughout the experiment, [^13^C_1_]acetic acid emissions remained low (below 5%). A similar abundance of [^13^C_2_]methyl acetate emissions was also observed with up to 85% of total emissions, while [^13^C]formic acid emissions reached 15% of the total.

### 2.6. ^13^C-Labeling Analysis of Isoprene (C_5_H_8_) Emissions and Its Dependence on ^13^CO_2_

During the delivery of the ^13^C_1_ and ^13^C_2_ solutions via the transpiration stream, the quantitative relationship between the production of isoprene, formed from the primary photosynthetic products, and internal CO_2_ produced from the C_1_ pathway ([^13^C]methanol, [^13^C]formaldehyde, [^13^C]formate), photorespiration ([2-^13^C]glycine), and respiration ([^13^C_2_]acetate) were evaluated. Simultaneous PTR-MS, GC-MS, and IRMS/CRDS measurements of branch headspace air during ^13^C-solution feeding via the transpiration stream enabled ^13^C-labeling analysis of isoprene (C_5_H_8_) emissions and its dependence on ^13^CO_2_ emitted into the headspace atmosphere (Figure 6 and Supplementary Figure S1). Across all ^13^C_1_ and ^13^C_2_ solutions used, a strong positive linear correlation emerged between the percentage of ^13^CO_2_ and isoprene emissions as [^13^C_1_]isoprene (Figure 6f). Of all solutions delivered via the transpiration stream, [^13^C]formate resulted in the strongest labeling of both CO_2_ (^13^CO_2_ up to 16.5%) and isoprene (up to 38.5% emitted as [^13^C_1_]isoprene). In contrast, [^13^C]methanol solutions resulted in the weaker labeling with ^13^CO_2_ as low as 1.2–7.8% with 12.2–23.8% of isoprene emitted as [^13^C_1_]isoprene. [^13^C_2_]acetate solutions also stimulated ^13^CO_2_ emissions (^13^CO_2_ 3.2–6.2% of total) together with enhanced [^13^C_1_]isoprene labeling (11.1–18.5% of total). Although isoprene emissions with 2 and 3 ^13^C-atoms were observed (e.g., [^13^C_2,3_]isoprene), emissions were always lower than isoprene emissions with only 1 ^13^C-atom ([^13^C_1_]isoprene). This same pattern of ^13^C-isoprene labeling, with [^13^C_1_]isoprene dominating emissions followed by [^13^C_2_]isoprene and [^13^C_3_]isoprene, was observed during [^13^C]formate, [2-^13^C]glycine, and [^13^C_2_]acetate labeling, and suggests a common mechanism (Figure 6). Thus, although both ^13^C-atoms of [^13^C_2_]acetate were quantitatively incorporated into [^13^C_2_]acetic acid and [^13^C_2_]methyl acetate emissions (Figure 5), [^13^C_1_]isoprene emissions were higher than [^13^C_2_]isoprene emissions (Figure 6).

### 2.7. Quantitative Comparisons of Emission Rates of C_1_ and C_2_ Volatiles

During [^13^C]methanol labeling experiments, emission rates of ^13^C-labeled C_1_ and C_2_ volatile intermediates were quantitatively compared with that of [^13^C]methanol emissions during steady-state conditions. After accounting for the number of ^13^C-atoms in each compound, 1.5% was emitted as [^13^C]formaldehyde, 0.3% was emitted as [^13^C]formic acid + [^13^C_2_]ethanol, 1.5% was emitted as ^13^CO_2_, and 0.2% was emitted as [^13^C_2_]acetic acid. Therefore, [^13^C]methanol emissions dominated emissions of ^13^C-labeled volatiles. Moreover, during [^13^C]formaldehyde labeling, [^13^C]methanol emissions again dominated emissions of ^13^C-labeled volatiles while [^13^C]formaldehyde emissions represented only a minor fraction. Relative to [^13^C]methanol emissions, 2.5% was emitted as [^13^C]formaldehyde, 0.5% as [^13^C]formic acid, and 3.8% as [^13^C_2_]acetic acid (note ^13^CO_2_ observations were not available during the [^13^C]formaldehyde labeling experiments). When [^13^C]formate labeling was applied, [^13^C]methanol and [^13^C]formaldehyde could not be detected and ^13^C-labeled volatile emissions were dominated by ^13^CO_2_. Relative to ^13^CO_2_ emissions, only 5.3 × 10^−4^% was emitted as [^13^C]formic acid and 5.3 × 10^−4^% was emitted as [^13^C_1_]acetic acid. A similar result was obtained during [2-^13^C]glycine labeling. Relative to ^13^CO_2_ emissions only 2.5 × 10^−3^% was emitted as [^13^C]formic acid and 5.0 × 10^−3^% was emitted as [^13^C_1_]acetic acid. This results demonstrate that under [^13^C]methanol or [^13^C]formaldehyde labeling, [^13^C]methanol dominates emissions while under [^13^C]formate or [2-^13^C]glycine labeling, the dominant emissions are ^13^CO_2_.

## 3. Discussion

### 3.1. Dynamics of Labeling between [^13^C]Methanol and [^13^C]Formaldehyde Solutions

After methane, methanol is the most abundant VOC in the global atmosphere with emissions tightly connected to plant growth. However, to date, it is generally assumed that methanol represents a byproduct of the expansion of cell walls during growth processes and that production rates over a day are equal to the total emissions. Although evidence for the existence of a C_1_ pathway in plants was first collected over 50 years ago, its intermediates are difficult to measure and relatively little is known about this potentially ubiquitous, yet mysterious biochemical pathway.

In this study, we employed the dynamic ^13^C-pulse chase technique under photorespiratory CO_2_ concentrations (100 ppm) to evaluate the existence of the C_1_ pathway and its integration with C_2_ metabolism in individual branches of a tropical tree species using aqueous solutions of ^13^C-labeled C_1_ (methanol, formaldehyde, formic acid) and C_2_ (acetic acid, glycine) intermediates delivered via the transpiration stream. [^13^C]methanol labeling resulted in the rapid production of each major volatile intermediate of the oxidative C_1_ pathway including [^13^C]formaldehyde, [^13^C]formic acid, and ^13^CO_2_, confirming that methanol initiates the complete C_1_ pathway (methanol, formaldehyde, formic acid, carbon dioxide) in *I. edulis* (Figure 1). Emission rate comparisons suggested that during [^13^C]methanol labeling, only a few percent of [^13^C]methanol emissions are emitted as [^13^C]formaldehyde, consistent with the view of formaldehyde as highly reactive and undergoes numerous transformations within cells including oxidation to formate, activation to 5,10-CH_2_-THF, and formaldehyde adducts with amino acids and proteins [25]. Consistent with this view, during [^13^C]formaldehyde labeling, [^13^C]formaldehyde emissions were 40 times lower than [^13^C]methanol emissions.

**Figure 7:**
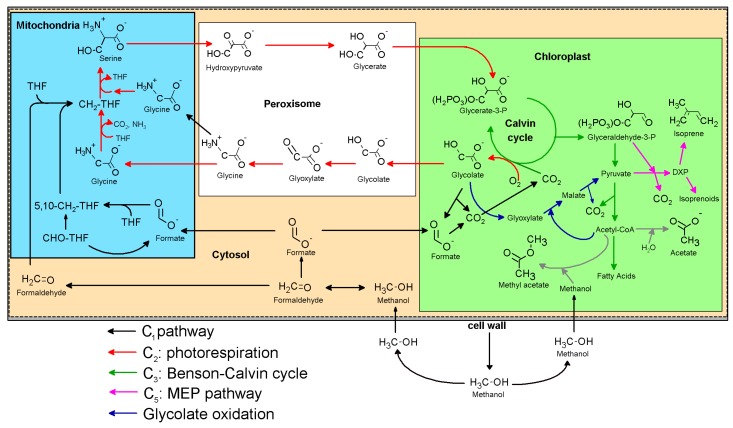
Simplified schematic model of the integration of the C_1_ pathway into C_2_, C_3_, and C_5_ metabolism in a photosynthetic plant cell. Note that the C_1_ pathway supports both photorespiration in mitochondria by producing 5,10-CH_2_-THF and photosynthesis in the chloroplast by producing CO_2_.

As methanol emissions have been observed from all tropical species studied [12], this finding suggests the C_1_ pathway is highly active in the tropics. The rapid disappearance of the ^13^C-label in the volatile C_1_ intermediates within minutes upon replacing the [^13^C]methanol solution with methanol demonstrates the rapid turnover of these intermediates within leaves under photorespiratory conditions. Although [^13^C]methanol solutions resulted in the strong labeling of [^13^C]formaldehyde emissions (Figure 1) and [^13^C]formaldehyde solutions resulted in the strong labeling of [^13^C]methanol emissions (Figure 2), [^13^C]formate solutions did not result in detectable [^13^C]methanol or [^13^C]formaldehyde emissions. The results suggest that the conversion between methanol and formaldehyde is reversible, whereas once formaldehyde oxidation to formate occurs, it is quickly oxidized to CO_2_ within chloroplasts where it can be re-assimilated by photosynthesis and contribute carbon to primary photosynthetic products like isoprene (Figure 7). Thus, when [^13^C]formate labeling was applied, ^13^CO_2_ emissions were hundreds of thousands of times greater than formic acid emissions (Figure 3), resulting in the strongest ^13^CO_2_ labeling observed (Figure 6).

### 3.2. Reassimilation of CO_2_ from C_1_ Intermediates and Its Contribution as Carbon Source for Isoprene

During ^13^C-labeling with C_1_ intermediates, significant labelling of CO_2_ and isoprene emissions were observed. However, the strongest incorporation of ^13^C-isoprene labelling was found in branches fed with [^13^C]formate (Figure 6). The high yield and velocity of transformation of [^13^C]formate to ^13^CO_2_ may be linked to the catalytic activity of formate dehydrogenase [29,30,31]. This enzyme has been demonstrated to play a key role in controling the formate pool size and redirection of C_1_ flux towards CO_2_ release in plants cells [32]. Its localization in chloroplasts [30] may favour the reincorporation of CO_2_ release from formate decarboxylation into photosynthetic products via the Benson-Calvin cycle [33]. This process was previously demonstrated by the incorporation of [^14^C]formate into sugars and amino acids in illuminated leaves, but not leaves in the dark [30]. Therefore, reassimilation of [^13^C]formate derived ^13^CO_2_ may explain the emissions of [^13^C_1_]isoprene as a primary photosynthetic product [34]. While formate may be oxidized directly to CO_2_, formaldehyde is highly reactive and has numerous biochemical fates [35]. This fact can explain the stronger ^13^C-labeling of CO_2_ and isoprene emissions with [^13^C]formate compared with [^13^C]methanol and [^13^C]formaldehyde solutions. This idea is supported with a previous study which found that formate oxidation to CO_2_ is the primary fate of formate in *Arabidopsis thaliana* [36]. The strong positive linear relationship between the percentage of ^13^CO_2_ and [^13^C_1_]isoprene, irrespective of the specific [^13^C]-labeled C_1_ or C_2_ substrate supplied (Figure 6f), support an important role for the reassimilation of C_1_, photorespiratory, and respiratory CO_2_, as ‘alternate carbon’ sources for photosynthetic products. Recent studies have suggested that internal recycling of CO_2_ may be a key mechanism for plant functioning under droughts and high temperatures when stomatal closure limits the uptake of atmospheric CO_2_ [37].

### 3.3. Integration of the C_1_ and C_2_ Metabolism

During [^13^C_2_]acetate labeling, emission rates of [^13^C_2_]acetic acid and [^13^C_2_]methyl acetate were strongly stimulated demonstrating the limitation of acetate/acetyl-CoA production for the emissions of acetic acid and methyl acetate to the atmosphere. Acetyl-CoA in chloroplasts is thought to derive mainly from plastidic pyruvate dehydrogenase activity [38]. For the first time in plants, we show that [^13^C]methanol and [^13^C]formaldehyde solutions delivered to the transpiration stream result in a rapid and quantitative turnover of carbon pools used in the biosynthesis of central C_2_ compounds (acetic acid and acetyl CoA). This process, analogous to the serine cycle in methyltrophic bacteria, may represent an important route for the biosynthesis of key C_2_ intermediates widely used in plant cells as precursors for a diverse suite of anabolic (e.g., fatty acid biosynthesis) and catabolic (e.g., mitochondrial respiration) processes. Methylotrophic microorganisms can utilize methanol as their sole carbon and energy source. In an analogy to photorespiration in plants, “methanol assimilation” in bacteria begins with the incorporation of C_1_ carbon into serine via the “serine cycle” resulting in the production of acetyl-CoA/acetate [27]. Thus, acetate is widely recognized as an important intermediate in microbial methanol assimilation and our observations suggest that a similar biochemical process may exist in tropical trees.

One possible mechanism for this quantitative C_1-2_ connection is the activation of formaldehyde to 5,10-CH_2_-THF, the methyl donor in the photorespiratory conversion of glycine to serine (Figure 7). Acetyl-CoA and formate may be produced from photorespiratory intermediates during glycolate oxidation [39,40]. However, as the glyoxylic acid cycle is generally considered active mostly in germinating seeds [41], the mechanism of acetate/acetyl-CoA production from C_1_ intermediates in plants deservers additional attention. However we note that quantitative conversion of carbon pools used in acetate/acetyl-CoA biosynthesis occurred during [^13^C]methanol and [^13^C]formaldehyde labeling and not during [^13^C]formate or [2-^13^C]glycine labeling, suggesting the a key role of 5,10-CH_2_-THF. Our observations are consistent with previous studies that demonstrated formaldehyde integrates into photorespiration in mitochondria by providing an alternate source of CH_2_-THF used for the methylation of serine to glycine, and operates independently from the Benson-Calvin cycle [21]. Earlier work supports this conclusion by demonstrating that formaldehyde carbon can be observed in amino acids [20] and later work showed a similar integration of formaldehyde carbon in sugars and organic acids [35]. Eliminating the need for a second glycine for CH_2_-THF production, the integration of the C_1_ pathway into photorespiration may convert it from a net loss of carbon to a net gain. By also suppressing photorespiration via the production of CO_2_ in chloroplasts, we hypothesize that the C_1_ pathway activity enhances carbon use efficiency, or the ratio of net photosynthesis to gross carbon assimilation.

Supporting this hypothesis, the application of foliar methanol sprays to cotton increased serine levels and decreased glycine levels [42]. Indeed previous research by one of the founding fathers of photosynthesis research (Dr. Andrew Benson), for whom this paper is dedicated, found evidence for an important role of methanol sprays in boosting plant photosynthesis, biomass, and productivity in agricultural crops [43,44]. However, this topic remains controversial as subsequent researchers were unable to observe these effects, and the biochemical mechanism(s) remain unclear. Nonetheless, these studies showed that providing methanol to C_3_ plants under stressful conditions can enhance plant productivity in crops, but fails to increase yield in C_4_ plants [43,44]. As C_3_ plants undergo more photorespiratory stress than C_4_ plants, these studies support the hypothesis that C_1_ compounds can help alleviate photorespiratory stress. However, several previous studies used very high aqueous concentrations of methanol (up to 40%). As natural aqueous concentrations of methanol in plant cells is expected to be much lower than that applied in methanol sprays and substantially lower than applied during the ^13^C-labeling studies presented here, the quantitative importance of the integration of the C_1_ pathway into central C_2_ metabolism, and its dependence on environmental and biological variables, remains an active area of research.

## 4. Materials and Methods

### 4.1. ^13^C Labeling Studies

This study was conducted in the experimental area and laboratory conditions of the V-8 Campus at the National Institute for Amazon Research (MCTI-INPA), Manaus-Brazil (3°5′35′′ S, 59°59′38′′ W). To study C_1_ metabolism and its integration into central carbon metabolism in plants, five naturally occurring 5–10 m tall *I. edulis* var. parviflora Benth. trees grown under field conditions were used. This species was selected because of its high isoprene emissions and because detached branches maintain high transpiration rates, and therefore readily take up the ^13^C-labeled metabolite solutions, for at least 12 h following branch detachment from the tree.

The stems of detached branches (2.7–3.2 g dry mass) were placed in ^13^C-labeled metabolite solutions and the leaves were sealed in a 4.0 L Teflon branch enclosure under constant light (300–500 µmol·m^−2^·s^−1^ photosynthetic photon flux density) and air temperature (28–30 °C). The flow-through of hydrocarbon free air was maintained at 2.0 L·min^−1^ using a pure air generator system (model 737, Aadco Instruments Inc., Cleves, OH, USA), which activated photorespiratory processes by producing sub-ambient concentrations of CO_2_ (100 +/− 10 ppm). Measurements of methanol, formaldehyde, formic acid, ethanol, acetic acid, methyl acetate, and isoprene were performed with PTR-MS and GC-MS, while CO_2_ labeling analysis was performed with an isotope ratio mass spectrometer (IRMS) or cavity ringdown spectrometer (CRDS) for isotopic CO_2_. Three to five replicate branch labeling experiments were performed on successive days for each of the following solutions; [^13^C]methanol (10 mM), [^13^C]formaldehyde (5 mM), [^13^C]sodium formate (10 mM), [2-^13^C]glycine (10 mM), and [^13^C_2_]acetate (10 mM).

### 4.2. Proton Transfer Reaction-Mass Spectrometry (PTR-MS)

Real-time emissions of C_1_, C_2_, and C_5_ volatile metabolites from detached *I. edulis* branches were carried out using a commercial high sensitivity proton transfer reaction mass spectrometer (PTR-MS, Ionicon, Innsbruck, Austria). The PTR-MS was operated under standard conditions with a drift tube voltage of 600 V and drift tube pressure of 200 Pa. Optimization of PTR-MS conditions resulted in high and sustained primary ion intensities (2–4 × 10^7^ counts per second for H_3_O^+^) with low water cluster (H_2_O-H_3_O^+^ < 4% H_3_O^+^) and O_2_^+^ (O_2_^+^ < 4% H_3_O^+^) formation. The following mass to charge ratios (*m*/*z*) were sequentially monitored during each PTR-MS measurement cycle; 21 (H_3_^18^O^+^) and 37 (H_2_O-H_3_O^+^) with a dwell time of 20 ms each. In addition, the following mass to charge ratios were sequentially monitored with 1.0 s dwell time each: 31 (formaldehyde-H^+^), 32 (O_2_^+^ + [^13^C]formaldehyde-H^+^), 33 (methanol-H^+^), 34 ([^13^C]methanol-H^+^), 43 (acetate fragment-H^+^), 44 ([^13^C]acetate fragment-H^+^), 45 (acetaldehyde-H^+^), 46 ([^13^C]acetaldehyde-H^+^), 47 (formic acid-H^+^ + ethanol-H^+^), 48 ([^13^C]formic acid-H^+^ + [^13^C]ethanol-H^+^), 49 ([^13^C_2_]ethanol-H^+^), 59 (acetone-H^+^), 60 ([^13^C]acetone-H^+^), 61 (acetic acid-H^+^), 62 ([^13^C]acetic acid-H^+^), 63 ([^13^C_2_]acetic acid-H^+^), 69 (isoprene-H^+^), 70 ([^13^C]isoprene-H^+^), 71 ([^13^C_2_]isoprene-H^+^), 72 ([^13^C_3_]isoprene-H^+^), 73 ([^13^C4]isoprene-H^+^), 74 ([^13^C5]isoprene-H^+^), 75 (methyl acetate-H^+^), 76 ([^13^C]methyl acetate-H^+^), 77 ([^13^C_2_]methyl acetate-H^+^), and 78 ([^13^C_3_]methyl acetate-H^+^).

Raw signals (counts per second, cps) were normalized by the adjusted primary ion signal (cps_21_) to obtain normalized counts per second (ncps = cps/cps_21_). The adjusted primary ion signal (cps_21_) was obtained by multiplying the signal at *m*/*z* 21 (H_3_^18^O^+^) by the oxygen isotopic ratio of a representative natural abundance water sample (^16^O/^18^O = 500).

### 4.3. Gas Chromatography-Mass Spectrometry (GC-MS)

Air samples were collected and analyzed for ^13^C-labeling of methyl acetate and isoprene using thermal desorption-gas chromatography-mass spectrometry (TD-GC-MS) at the National Institute of Amazonian Research (INPA) in Manaus, Brazil. Methyl acetate and isoprene volatiles in the branch enclosure air samples were collected by drawing 100 mL·min^−1^ of enclosure air through a TD tube for 5 min (isoprene) or 30 min (methyl acetate) by connecting a mass flow controller and a pump downstream of the TD tube. Inert coated stainless steel TD tubes were purchased commercially, filled with Quartz wool, Tenax TA, and Carbograph 5TD adsorbents (Markes International, Llantrisant, UK). The TD tube samples were analyzed for methyl acetate and isoprene with a thermal desorption system (TD-100, Markes International, Llantrisant, UK) interfaced with a gas chromatograph/electron impact mass spectrometer with a triple-axis detector (5975C series, Agilent Technologies, Santa Clara, CA, USA). The GC-MS was calibrated to methyl acetate with an authentic methyl acetate standard (99%, Sigma Aldrich, St. Louise, MI, USA) using dynamic solution injection (DSI) technique [45] and to isoprene through dynamic dilution of a commercial gas phase isoprene standard (1.0 ppm in N_2_, Apel-Reimer Environmental Inc., Broomfield, CO, USA) with high purity zero air.

After loading sample tubes in the TD-100 thermal desorption system, the samples collected were dried by purging for 10 min with 20 mL·min^−1^ of ultra-high purity helium (all flow vented out of the split vent) before being transferred (300 °C for 10 min with 20 mL·min^−1^ of helium) to the TD-100 cold trap (Air Toxics, Markes International, UK) held at 20 °C. During GC injection, the trap was heated to 300 °C for 3 min while back-flushing with carrier gas at a flow of 3.5 mL·min^−1^ with 2.0 mL·min^−1^ of this flow was directed to the split and 1.5 mL·min^−1^ directed to the column (Rtx 624 with intraguard, 60 m + 1 m guard × 0.32 mm × 1.8 µm, Restek Inc., Bellefonte, PA, USA). The oven temperature was programmed with an initial hold of 3 min at 35 °C followed by an increase to 230 °C at 6 °C·min^−1^. The mass spectrometer was configured for trace analysis with a 15 times detector gain factor and operated in either scan mode for isoprene analysis (*m*/*z* 24–100) or selected ion mode for methyl acetate analysis (*m*/*z* 31, 32, 43, 44, 45, 59, 60, 61, 74, 75, 76, 77).

### 4.4. CO_2_ Isotope Measurements

During some of the pulse-chase experiments with positional specific ^13^C-labeled metabolites, continuous online measurements of CO_2_ mixing ratios and carbon isotopic composition of CO_2_ were made with a cavity ring-down spectrometer (CRDS, G2101-i, Picarro Inc., Santa Clara, CA, USA), which enables real-time analysis of the isotopologues ^13^CO_2_ and ^12^CO_2_ as well as the δ^13^CO_2_ ratio (‰) of the sample gas relative to the factory calibrated reference standard Vienna PeeDee Belemnite (VPDB). For a single *I. edulis* branch supplied with [^13^C]methanol, carbon isotope ratios of CO_2_ in the enclosure headspace was measured via direct injection into the inlet of a Tracegas™ pre-concentrator interface associated with a Micromass JA Series Isoprime isotope ratio mass spectrometer (TG-IRMS, Micromass, Manchester, UK). The gas samples were injected into an evacuated inlet tube (~400 Pa) using a gas tight syringe. By flushing with ultra-pure helium the sample was transferred to liquid nitrogen traps. By heating the traps, the pre-concentrated CO_2_ was transferred and chromatographically separated on a PoraPLOT Q fused silica capillary column (30 m × 0.32 mm), and the carbon isotope ratio was measured in the mass spectrometer. Carbon isotope ratios are reported in the conventional δ-notation relative to VPDB scale. Repeated injections of CO_2_ associated with sample analysis yield values of −39.12 ± 0.40‰ (±SD; *n* = 8).

## 5. Conclusions

Methanol is highly abundant in the global atmosphere and is known to be tightly connected to plant growth. However, to date, it has been assumed that methanol represents a byproduct of the expansion of cell walls during growth processes. Although evidence for the existence of a C_1_ pathway in plants was first collected over 50 years ago, little is known about this potentially ubiquitous biochemical pathway, in part due to the high volatility of several key intermediates. While the mechanism(s) for the quantitative carbon connection between C_1_ and C_2_ metabolism require additional investigation, our study supports a direct incorporation of C_1_ carbon into central carbon and energy metabolism (Figure 7).

In this study, we employed dynamic ^13^C-pulse chase labeling to evaluate the potential existence of the complete C_1_ pathway and its integration with C_2_ metabolism in individual branches of a tropical pioneer species using aqueous solutions of ^13^C-labeled C_1_ (methanol, formaldehyde, formic acid) and C_2_ (acetic acid, glycine) intermediates delivered via the transpiration stream. The results confirm that methanol initiates the complete C_1_ pathway in plants (methanol, formaldehyde, formic acid, carbon dioxide) by providing real-time dynamic ^13^C-labeling data showing their interdependence. Also evident is the rapid interconversion between methanol and formaldehyde, whereas once oxidation to formate occurs, it is quickly oxidized to CO_2_ within chloroplasts where it can be re-assimilated by photosynthesis and therefore contribute to photosynthetic products like isoprene. We show that reassimilation of C_1_, respiratory, and photorespiratory CO_2_ is a common mechanism for isoprene biosynthesis; a strong linear dependence of ^13^C-labeling of isoprene on ^13^C-labeling of CO_2_ was observed across all C_1_ and C_2_^13^C-labeled substrates. Finally, we show, for the first time, that methanol and formaldehyde delivery to the transpiration stream leads to a rapid and quantitative conversion of carbon pools used in the biosynthesis of central C_2_ compounds (acetic acid and acetyl CoA) and therefore represents a potentially new uncharacterized source of these key C_2_ intermediates widely used as precursors for a diverse suite of anabolic (e.g., fatty acid biosynthesis) and catabolic (e.g., mitochondrial respiration) processes.

Our observations are consistent with previous studies that demonstrated formaldehyde integrates into photorespiration in the mitochondria by providing an alternate source of CH_2_-THF used for the methylation of serine to glycine. By eliminating the need for a second glycine for the production of CH_2_-THF with the subsequent loss of CO_2_ and NH_3_, the integration of C_1_ pathway into photorespiration may convert it from a net loss of carbon to a net gain. By also suppressing photorespiration via the production of CO_2_ in chloroplasts, our study presents the hypothesis that the integration of C_1_ pathway into C_2/3_ metabolism may boost carbon use efficiency and therefore represent an important mechanism by trees under photorespiratory conditions (e.g., high temperature stress). As agricultural crops are known to be high methanol producers, genetic manipulation of the C_1_ pathway has the potential to improve yields and tolerance to environmental extremes, thereby providing a new tool to the agriculture, bioenergy, and biomanufacturing industries.

## Supplementary Materials

Supplementary materials can be found at www.mdpi.com/1422-0067/18/10/2045/s1.

## Dedication

This paper is dedicated to Andrew Benson, a distinguished emeritus professor of biology at Scripps Institution of Oceanography, UC San Diego, and renowned as one of the world’s leading plant scientists of the twentieth century. Andy Benson’s pioneering work using ^14^CO_2_ led to the deciphering of the pathway of carbon fixation in photosynthesis. By initiating research into the stimulation of plant growth by alcohols, Andy laid the foundation for the discovery of the integration of C_1_ and C_2_ metabolism in plants. A website dedicated to the life of Andy Benson has been established (https://scripps.ucsd.edu/news/obituary-notice-andrew-benson-world-renowned-scripps-plant-biochemist).
