# Supplementary material for: Integration of C1 and C2 Metabolism in Trees
Source: Int J Mol Sci. 2017 Sep 23;18(10):2045. doi: 10.3390/ijms18102045 (PMC5666727; doi:10.3390/ijms18102045)
Supplement: Supplementary file 1 [file ijms-18-02045-s001.pdf]

International Journal of
Molecular Sciences

MDPI

Article

Integration of C_1 and C_2 Metabolism in Trees

Kolby J. Jardine [1,*], Vinicius Fernandes de Souza [2], Patty Oikawa [3], Niro Higuchi [2], Markus Bill [1], Rachel Porras [1], Ülo Niinemets [4,5] and Jeffrey Q. Chambers [1,6]

1 Climate Science Department, Earth Science Division, Lawrence Berkeley National Laboratory, One Cyclotron Rd, building 64-241, Berkeley, CA 94720, USA; mbill@lbl.gov (M.B.); rcporras@lbl.gov (R.P.); jchambers@lbl.gov (J.Q.C.)
2 National Institute for Amazon Research, Ave. Andre Araujo 2936, Campus II, Building LBA, Manaus AM 69.080-97, Brazil; viniciusfernandes11@yahoo.com.br (V.F.d.S.); higuchi.niro@gmail.com (N.H.)
3 Department of Earth and Environmental Sciences, California State University, East Bay, North Science 329, 25800 Carlos Bee Boulevard, Hayward, CA 94542, USA; patty.oikawa@csueastbay.edu
4 Department of Plant Physiology, Estonian University of Life Sciences, Kreutzwaldi 1, 51014 Tartu, Estonia; ylo@emu.ee
5 Estonian Academy of Sciences, Kohtu 6, 10130 Tallinn, Estonia
6 Department of Geography, University of California Berkeley, 507 McCone Hall #4740, Berkeley, CA 94720, USA
* Correspondence: kjjardine@lbl.gov; Tel.: +55-(92)-99200-7280

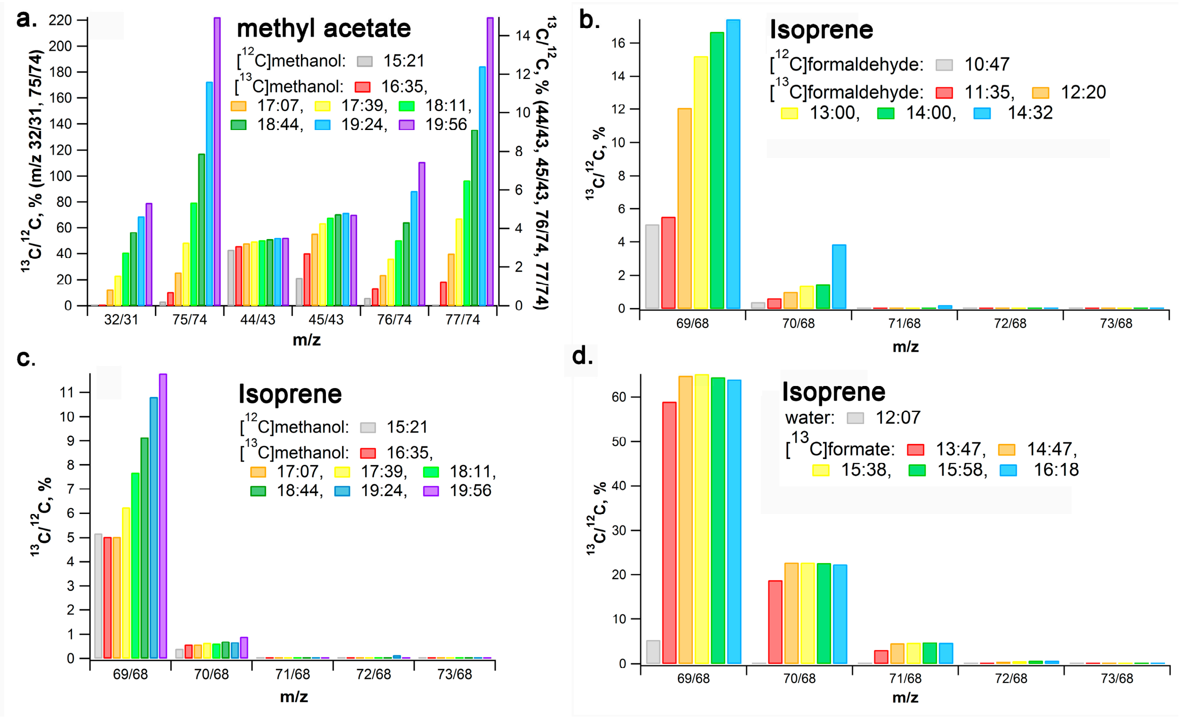

Figure S1. Example time series of 13C/12C ratios of methyl acetate (a) and isoprene (b-d) emissions determined by thermal desorption GC-MS during continuous [13C]methanol, [13C]formaldehyde, and [13C]formate labeling of an I. edulis branch.

© 2017 by the authors. Submitted for possible open access publication under the terms and conditions of the Creative Commons Attribution (CC BY) license (http://creativecommons.org/licenses/by/4.0/).
